# A fragment of Foley catheter balloon as a cause of Bladder stone in woman

**DOI:** 10.11604/pamj.2015.21.284.6770

**Published:** 2015-08-13

**Authors:** El Majdoub Aziz, Mouad Amrani, Khallouk Abdelhak, Farih Moulay Hassan

**Affiliations:** 1 Department of Urology, Hassan II Hospital University Center, Fez, Morocco

**Keywords:** intravésical, foreign body, catheter balloon, recurrent urinary tract infections

## Abstract

Urinary bladder calculi are rarely seen in women and any history of previous pelvic surgery must, therefore, raise suspicion of an iatrogenic etiology. According to the literature, fewer than 2% of all bladder calculi occur in female subjects and, thus, their presence should provoke careful assessment of the etiology. We report one case of a fragment of Foley catheter balloon as a cause of Bladder stone in 28 years old woman. Weanalyzed the diagnosis, aspect and therapeutic management of this case which is the first described in literature to our knowledge.

## Introduction

The bladder stones in woman are always secondary. Their presence should provoke careful assessment of the etiology in particular a foreign bodies. The formation of a bladder calculus around a foreign body raised astonishment on the introduction circumstances and the patient's psychological profile. We report one case of bladder stone including a fragment of Foley catheter balloon in a 28 years old woman, caused by an intravésical accidental breakage of balloon before the time of Caesarean section.

## Patient and observation

**Figure 1:**
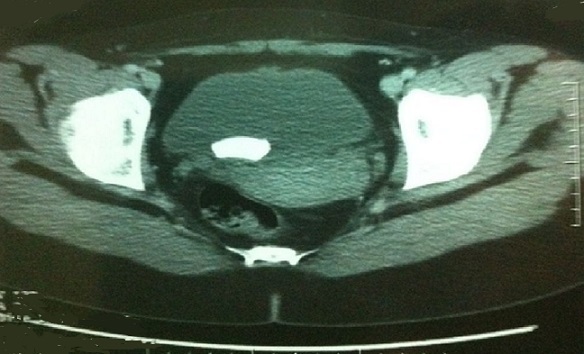
Pelvic computed tomography shows bladder stone (2 cm)

**Figure 2:**
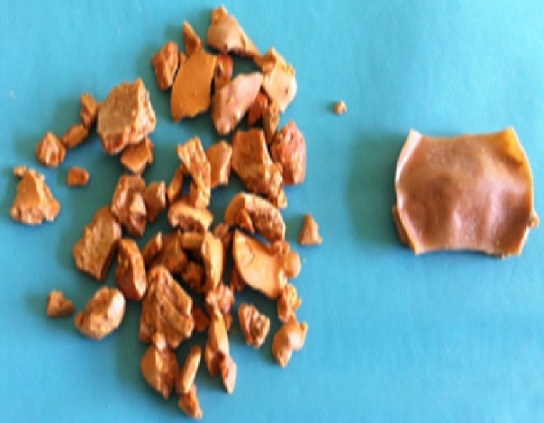
Bladder calculi formed around a fragment of Foley catheter balloon after intravesical lithotripsy

A 28-year-old woman presented with recurrenturinary tract infections despite many courses of antimicrobial therapy, frequency and burning urination since one year. The patient had a history of intravésical accidental breakage of Foley catheter balloon before the time of Caesarean section two years ago. She had no other medical problems. The physical examination was without abnormalities. ECBU showed a urinary tract infection treated by ceftriaxone. Other Laboratory examinations were normal. The abdominal radiograph showed a stone projecting on pelvis.ultrasonography and abdominal Computed tomography scan showed the presence of intravésical stone with high density, measuring 2,5 cm of diameter (Figure 1). The upper urinary tract was normal. Ballistic lithotripsy of the bladder stone with endoscopic extraction was then performed.in intraoperative we found a fragment of Foley probe balloon into the calculus (Figure 2).

## Discussion

Urinary bladder calculi are rarely seen in women and any history of previous pelvic surgery must, therefore, raise suspicion of an iatrogenic origin. According to the literature, fewer than 2% of all bladder calculi occur in female subjects and, thus, their presence should provoke careful assessment of the etiologyin particular foreign bodies. The presence of foreign bodies in the bladder arises the question of the mechanism of introduction. This mechanism can be iatrogenic during endoscopic surgery (broken equipment) or open transvesical open surgery (forgotten catheter, compress) or related to a voluntary psychiatric disease [1–3]. The frequency of foreign bodies in the bladder is difficult to specify. The textilomes represent 80% of those foreign bodies [4].the clinical manifestations that lead patients to consult are variable and nonspecific. They include urinary frequency, urge incontinence, voiding burns and variable intensity cystalgia. when the foreign body engages in the bladder neck it causes dysuria or acute retention of urine. Physical examination is often poor.the psychiatric profile should be precised. The abdominal radiograph may show calcifications projecting on the vesical area and also the foreign body if it is radio-opaque because it is often masked by a calcic stone. The ultrasonography showed hyperechoic images with posterior cone of shadow. While computed tomography urography is rarely requested, except when a repercussion on the upper urinary tract is suspected. In our case the fragment of Foley catheter balloon is unknown, which formed a nidus for secondary vesical calculus formation. It's acceleratedby recurrent urinary tract infections. A scan of the literature showed that it is the first case of this this type of bladder foreign body. The only presence of a foreign body in the bladder is sufficient to cause the formation of a calculus by promoting the precipitation of crystals and also maintaining urinary tract infection responsible for vesical irritation and pyuria. The extraction of the calculus byendoscopic ways the treatment of choice; it is sometimes preceded by an endo-bladder lithotripsy [5]. The large stones require open surgery. Our Report suggests that all physicians must verify the integrity of the bladder catheter balloon when intravésical accidental breakage of balloon is happen and if it is the case it will be necessary to do a cystoscopy to extract possible fragment.

## Conclusion

The bladder stones in woman are always secondary. Their presence should provoke careful assessment of the etiology in particular a foreign bodies. Intravésical accidental breakage of balloon can be a cause of formation bladder calculus if a fragment is forgotten for a long time in the bladder.all physicians must verify the integrity of the bladder catheter balloon when this accident is happen to avoid this formationand if it is the case it will be necessary to do a cystoscopy to extract possible fragment.
